# Mothers’ and Children’s Mental Health During the COVID-19 Pandemic Lockdown: The Mediating Role of Parenting Stress

**DOI:** 10.1007/s10578-021-01230-6

**Published:** 2021-08-21

**Authors:** Alessandra Babore, Carmen Trumello, Lucia Lombardi, Carla Candelori, Antonio Chirumbolo, Elena Cattelino, Roberto Baiocco, Sonia Monique Bramanti, Maria Luisa Viceconti, Silvia Pignataro, Mara Morelli

**Affiliations:** 1 Department of Psychological, Health, and Territorial Sciences, University “G. d’Annunzio” of Chieti-Pescara, Chieti, Italy; 2 Department of Psychology, Sapienza University of Rome, Rome, Italy; 3 Department of Human and Social Sciences, University of Valle d’Aosta, Aosta, Italy; 4 Department of Developmental and Social Psychology, Sapienza University of Rome, Rome, Italy; 5 Department of Dynamic and Clinical Psychology, Sapienza University of Rome, Rome, Italy

**Keywords:** COVID-19, Child depression, Parenting stress, Maternal distress, Mother–child relations

## Abstract

The present study, carried out during the first peak of the COVID-19 outbreak in Italy, aimed at investigating the mental health of mothers and children during the nationwide lockdown. More specifically, the study investigated children’s depression and mothers’ individual distress and parenting stress, in comparison with normative samples. The mediating effect of mothers’ parenting stress on the relationship between mothers’ individual distress and children’s depression was also explored. Finally, the study analyzed whether children’s biological sex and age moderated the structural paths of the proposed model. A sample of 206 Italian mothers and their children completed an online survey. Mothers were administered self-report questionnaires investigating individual distress and parenting stress; children completed a standardized measure of depression. Mothers’ individual distress and parenting stress and children’s depression were higher than those recorded for the normative samples. Mothers’ parenting stress was found to mediate the association between mothers’ individual distress and children’s depression. With respect to children, neither biological sex nor age emerged as significant moderators of this association, highlighting that the proposed model was robust and invariant. During the current and future pandemics, public health services should support parents—and particularly mothers—in reducing individual distress and parenting stress, as these are associated with children’s depression.

## Introduction

Over the past year, the unexpected stressful event of a global pandemic has tested families across the world. At the time of writing (April 2020), the coronavirus disease 2019 (COVID-19) has spread from China to other countries, resulting in more than 30 million recorded cases, globally [1]. Italy was one of the first countries to have been severely affected by the pandemic, with the initial outbreak in February 2020 in a small town in Lombardy region (northern Italy). Over a short period, the virus spread to other Italian regions, resulting in a state of emergency. This led the government to lock down the entire country, in order to contain the number of victims and prevent a collapse of the healthcare system.

Since then, the pandemic has upset lives in many ways, with differential effects on various age groups. Most likely, children have particularly suffered from the pandemic’s long-term psychological, social, and economic impacts [2]. As suggested by Bronfenbrenner’s socioecological model [3], interactions between individuals and their environment may significantly affect human development. Bronfenbrenner’s theory analyzes child development at four levels: (a) the microsystem level, which represents the child’s most proximal environment (e.g., family, school, neighborhood, peer group); (b) the mesosystem level, which refers to relations between microsystems; (c) the exosystem level, which includes the indirect environment (e.g., a parent’s workplace, the government, mass media), and (d) the macrosystem level, which represents the sociocultural environment (e.g., healthcare policy, society, values). In the outbreak context, the effects of the pandemic on the latter three levels (namely, mesosystem, exosystem and macrosystem) may be important risk factors for children’s microsystem [2].

In Italy, the government restrictions during the first lockdown involved the closure of all facilities (including schools and universities) and public spaces, except for those providing basic necessities (e.g., healthcare services and food providers); a stay at home order outside of situations of necessity; and the isolation of persons infected by COVID-19 and those in contact with them [4]. This critical situation upset family routines [5] by prohibiting all engagement with most activities, friendships, and relational contacts outside of the home. Suddenly, parents had to manage their children at home all day with no external help (from, e.g., grandparents, babysitters, educators). At the same time, most parents had to cope with job stress (e.g., risk of redundancy or salary reductions) and adjust to so-called “smart-working from home.” Many parents also had to manage difficulties and pain related to sick or dying relatives. All of these aspects are likely to have impacted parents’ distress levels, with a possible impact on children’s well-being [6, 7].

Parents’ distress may refer to individual factors (e.g., job difficulties, concern for relatives) or factors associated with relationships with children. The former refer to subjective experiences of stress [8] and do not specifically concern child-rearing, but are attributable to the wider social and environmental context, including everyday demands and responsibilities [9, 10]. The latter are associated with “parenting stress”—a particular type of stress related to the parental role. Parenting stress is usually described as parents’ negative experience, resulting from a perceived disparity between their parental responsibilities and their available resources [11, 12]. It tends to manifest in three concerns: (1) belief that one is not adequate in the parental role, (2) the attribution of an internal negative quality to the child, and (3) assessment of one’s interactions with the child as dysfunctional [13]. In the context of COVID-19, “parenting stress related to the parent–child relationship” [14] represents a particularly interesting factor, especially with regard to mothers, as, especially in Italy, mothers are saddled with the greatest burden of childcare [15]. In fact, the social distancing measures associated with the pandemic (and related changes to work, school, and childcare routines) have forced many mothers to develop new routines and set new limitations with their children. Research has shown that, when children are not at school, they suffer from greater boredom, they are less physically active, and they spend more time in front of screens [16], thereby increasing the likelihood for conflict with parents. In this situation, parents may have less control over their children’s maintenance of healthy behaviors [17], and they may find it necessary to re-negotiate rules with their children that, prior to the pandemic, were less difficult to manage (e.g., sharing common spaces, using smartphones/devices) or even non-existent (e.g., restrictions on outside activities). For example, under normative circumstances, time spent using technology is typically an “arena of conflict” between children and parents [18, 19]. During the lockdown, use of electronic devices, screen time, and sedentary activities increased among children and adolescents [20, 21], making this already contentious arena more difficult for parents to manage.

The current study focused on parenting stress related to the parent–child relationship. Parents with higher perceived parenting stress tend to be less sensitive to their children’s needs [11] and to show more dysfunctional interactions with their children [22]. Recent research [23, 24] has also confirmed that parenting stress is associated with a series of maladaptive child outcomes [11, 25], even over the long term [26].

As stated above, parental stress may also arise in association with factors that are external to the relationship with the child. Research on previous pandemics has reported an association between quarantine and high psychological distress, including depression, anxiety, and post-traumatic stress symptoms [27, 28], with long-lasting outcomes [29]. Parents’ individual distress has also been associated with poor mental health in children [30, 31]. Specifically, depression and anxiety in mothers are risk factors for depressive and anxiety symptoms in children [32, 33], with effects from early to middle childhood [33, 34] and adolescence [35]. Depressed mothers also present greater difficulties in parental behavior, including more negative interactions and more inconsistent responses to children’s needs [36]. Furthermore, mothers’ depressive symptoms may increase children’s likelihood of experiencing irritability, depression, anxiety, and learning problems [37, 38].

With respect to children, depressive symptoms are more prevalent from middle childhood onwards [39], and their intensity may relate to many environmental and individual factors [40]. As for the former, the quality of the maternal relationship and the mother’s emotional availability are important aspects [41]. With regard to individual features, age and biological sex are relevant; specifically, both the female gender and older ages are associated with higher levels of depression [42, 43]. Finally, a major risk factor for depression in children is parental depression—particularly maternal depression [44]—which may generate dysfunctional parent–child interactions that negatively influence children’s psychological status [45]. With regard to the current pandemic, a recent review highlighted a strong association between loneliness and social isolation with depression in children and adolescents [46]. As children and adolescents were isolated from their friends, classmates, teachers, and other relevant adults during the lockdown, particular attention should be paid to their depressive symptoms. In a recent study comparing depressive and anxiety levels before and during the COVID-19 lockdown, a significant increase in depressive symptoms—but not in anxiety—was observed [47]. Another study, conducted during the first peak of the COVID-19 pandemic, obtained similar results of increased depression and reduced anxiety [48, 49].

The presence of sensitive caregivers can represent an important protective factor [50] against inappropriate responses to stressful events. In fact, stressful life events, such as natural disasters, a parent’s serious illness, or worsening economic conditions, can increase parental stress, with significant repercussions for the well-being of both parents and children [51, 52]. Few studies have specifically investigated the associations among mothers’ individual distress, mothers’ parenting stress, and child depression. In a recent study, Tsotsi et al. [53] found that maternal parenting stress mediated the association between maternal anxiety and children’s psychological problems. However, to our knowledge, this relationship has not been investigated during the current pandemic, which represents an extreme situation that is likely to have affected the well-being of both mothers and children.

Consistent with the socioecological model of human development [3], the present study aimed at investigating the mental health of mothers and children during the first peak of the COVID-19 pandemic in Italy. As suggested by a recent study [2], the impact of the pandemic on the complex interactions among Bronfenbrenner’s meso-, exo- and macrosystem levels may represent risk factors for the microsystem of the family and child and the mental health of parents and children. Therefore, following the recent suggestion of Jiao et al. [54] to widen the literature on children’s responses to epidemics, the first aim of the present study was to compare children’s depression, mothers’ individual distress, and mothers’ parenting stress to normative levels. We expected that the mean scores in the present sample would be significantly higher than those registered by the normative population, given the stressful situation related to the health emergency within Brofenbrenner’s macrosystem. Second, we hypothesized a bivariate significant association between mothers’ individual distress, mothers’ parenting stress, and children’s depression. In addition, given the scarcity of research exploring the potential mechanisms underlying the association between mothers’ individual distress related to the pandemic and child depression, we examined whether parenting stress might serve as the mediating variable. Specifically, within the framework of Bronfenbrenner’s socioecological model [3], we aimed at examining how the trauma of the pandemic in the macrosystem [2], as reflected in parenting stress due to the health emergency, impacted the microsystem, in terms of mothers’ individual distress and its effect on children’s depression. Therefore, we tested a path model in which an indirect effect of mothers’ individual distress on child depression, mediated by mothers’ parenting stress, was expected. In other words, we hypothesized that the effect of mother’s individual distress (the predictor variable) on child depression (the outcome variable) would be conveyed by mothers’ parenting stress (the mediator). Finally, we evaluated whether children’s biological sex and age were potential moderators of the proposed model. We thus tested the possible moderation effects of age and gender on the relationships among the investigated variables, because previous studies have shown a possible effect of children’s dependence on caregivers. However, these studies have presented inconsistent results: some have shown that the parents of children (versus adolescents) have similar levels of parenting stress [55]; other studies have found that parenting stress increases with child age [56].

## Methods

### Participants and Procedure

Two a priori power analyses were conducted to determine the recommended minimum sample size for: (1) detecting a significant bivariate effect and (2) conducting a structural equation model (SEM) [57, 58]. A moderated effect size of 0.25 was anticipated with power level set at 0.80 and a significant alpha level set at 0.05. The minimum sample size necessary to detect a significant bivariate effect was *N* = 124. As regards the SEM, it considered 3 latent and 12 observed variables. Considering these parameters, using the software developed by Soper [59], the results indicated that the required minimum sample size to run a SEM and detect a significant effect was *N* = 181.

A total of 206 Italian mothers, aged 27–60 years (*M* = 43.87; *SD* = 5.95), and their children, aged 7–18 years (*M* = 12.18; *SD* = 3.31; 50.5% girls), completed an online survey during April 2020, representing the first peak of the pandemic in Italy, when a nationwide lockdown was in place. School-age children were selected for two main reasons: (1) at this age, children are able to independently answer questionnaires [60] and (2) depressive symptoms tend to increase during late childhood and early adolescence [39, 61, 62].

A web-based survey, developed using Qualtrics XM, was distributed over the Internet via mainstream social networks (WhatsApp, Facebook, etc.). In addition, parents were contacted directly and indirectly. For example, we contacted the headmasters of schools, asking if they might circulate the survey link to parents; we posted the survey link on some Facebook groups for parents, inviting participants with children aged 8–18 years; we asked teachers to share the survey link on their parent WhatsApp groups; and we contacted the presidents of some voluntary associations that deal with children, asking them to share the survey link on their parents WhatsApp groups. Thus, participants were recruited via snowball sampling and asked to forward the survey to other parents. Mothers reported their individual distress and parenting stress. They also gave their consent to participate in the study on the first page of the online survey; once they completed the questionnaire, they were asked to give consent for their children to participate in the second part of the study. Children who were aged 18 years gave their own consent to fill in the survey. Those who agreed to participate completed a questionnaire that was designed to investigate their depressive symptoms. Overall, 503 mothers of children aged 7–18 years participated in the study; 386 (76.74%) gave consent for their child to participate; 212 children (212/386 = 54.92%) filled in part of the questionnaire and 206 (53.37%) completed the questionnaire in full. Mothers who consented to their child’s participation (*N* = 386) and mothers who did not give consent (*N* = 117) were compared with respect to age, parenting stress, and individual distress. No significant differences were found between groups regarding mothers’ age, *t*(501) =  − 0.42, *p* = 0.67, parenting stress, *t*(501) =  − 0.89, *p* = 0.37, or individual distress, *t*(501) =  − 0.30, *p* = 0.76. Furthermore, among the mothers who consented to their child’s participation, we compared those whose child completed the questionnaire and those whose child did not complete the questionnaire. No significant differences were found between groups regarding mothers’ age, *t*(384) =  − 1.47, *p* = 0.14, parenting stress, *t*(384) =  − 0.53, *p* = 0.60, or individual distress, *t*(384) = 0.70, *p* = 0.48. When completing the questionnaire, mothers and children were asked to refer to the lockdown period, only. Fifty-four mothers were from northern Italy (26.2%), 35 were from central Italy (17%), and 117 were from southern Italy (56.8%). Regarding mothers’ economic status, 5 (2.4%) reported that it had improved, 106 (51.5%) reported no change, and 95 (46.1%) reported worsening. The study was approved by the Ethics Committee of the University (blinded for review).

### Measures

Sociodemographic data for mothers (with respect to age, education, and socioeconomic and employment status) and children (with respect to age and gender) were collected.

#### Mothers’ Individual Distress

Individual distress in mothers was assessed using the Hospital Anxiety and Depression Scale (HADS) [63, 64]—a self-report instrument consisting of 14 items. Items are rated on a 4-point Likert scale ranging from 0 to 3 (sample items: “Worrying thoughts go through my mind”; “I can laugh and see the funny side of things”). The HADS is usually employed to assess anxious and depressive symptoms related to the hospitalization of clinical patients [64]. A recent study also used the HADS to evaluate Italian parents’ anxiety and depression related to the COVID-19 pandemic [65]. The total score ranges from 0–42, with higher scores indicating higher distress. Previous studies have shown the HADS to have good psychometric properties [64, 65]. In the present study, the scale had good reliability, with Cronbach’s alpha = 0.88.

#### Parenting Stress

Consistent with our association of parenting stress with the parent–child relationship, and in line with previous studies [14, 66], we evaluated parenting stress using the Parent–Child Dysfunctional Interaction (P-CDI) scale of the Parenting Stress Index-Short Form (PSI-SF) [11, 67]—a 36-item self-report questionnaire. The PSI-SF has been administered to Italian parents of children [24] and adolescents [68], showing good psychometric properties. The P-CDI scale consists of 12 items (e.g., “My child rarely does things for me that make me feel good”), scored on a 5-point scale ranging from “strongly agree” to “strongly disagree.” The questionnaire assesses parental perceptions of the emotional quality of interactions with children, particularly with respect to dissatisfaction. Higher scores are associated with higher parenting stress. The Italian validation of the PSI-SF showed it to have good internal consistency [67]. In the present study, the P-CDI scale achieved good reliability, with Cronbach’s alpha = 0.89.

#### Children’s Depression

To assess depressive symptoms in children, we used the Patient Reported Outcomes Measurement Information System (PROMIS)–Emotional Distress-Depression-Pediatric Item Bank [69–71]—a self-report instrument focusing on negative mood (e.g., sadness), anhedonia (e.g., loss of interest), negative views of the self (e.g., worthlessness, low self-esteem), and negative social cognition (e.g., loneliness, interpersonal alienation). The PROMIS was designed to assess depressive symptoms in children and adolescents. It is commonly used for initial assessments in a clinical context and for monitoring symptoms during psychological treatment [71, 72]. The PROMIS encompasses 14 items (e.g., “I could not stop feeling sad”), rated on a 5-point Likert scale ranging from 1 (*never*) to 5 (*always*). Total scores range from 14 to 70, with higher scores indicating higher levels of depression. Previous studies have shown the PROMIS to have good psychometric properties [71, 73–75]. In the present study, the scale achieved excellent reliability, with Cronbach’s alpha = 0.91.

### Data Analysis

We first computed the descriptive statistics and correlations among variables. The first analysis investigated the correlations among variables and compared mothers’ individual distress, mothers’ parenting stress, and children’s depression scores to Italian normative means using a one-sample t-test. Published normative means were derived for the non-medical, non-psychiatric Italian population for the different scales [67, 71, 76].

Second, we tested the hypothesized mediational model by conducting a path analysis with latent variables using the software Mplus 8.3. Mediation analysis with latent variables was performed via SEM, utilizing a parceling strategy (e.g., [77–80]). A parcel denotes an aggregate of different items measuring a specific construct [79, 81]. Each parcel was constructed applying the “item-to-construct” balance approach [79], considering factor loadings in the item-level factor analyses representative of the item–construct relationships (for a detailed description of this procedure, see Little et al. [79]). With this approach, parcels typically include a balanced number of items with similar reliabilities. For the purpose of identification, it is usually recommended to have at least three observed variables for each latent variable [78]. Therefore, our final model comprised three latent variables: mothers’ parenting stress and children’s depression were measured with three parcels each, while mothers’ individual distress was assessed with six parcels.

Following the multifaceted approach for model fit evaluation in SEM [82], four indices were considered: (a) the comparative fit index (CFI), (b) the Tucker–Lewis index (TLI), (c) the root mean squared error of approximation (RMSEA), and (d) the standardized root mean square residual (SRMR). In general, TLI and CFI values between 0.90 and 0.95 are considered acceptable and values above 0.95 are deemed very good; in contrast, RMSEA and SRMR values smaller than (or equal to) 0.08 indicate good fit (e.g., [78, 83–86]).

Two nested models were tested: the first comprised a full mediated model (M1) in which the effect of mothers’ individual distress on children’s depression was completely mediated by parenting stress. The second represented a partial mediated model (M2) in which the effect of mothers’ individual distress on children’s depression had a significant direct effect. To decide which model to retain, the Satorra–Bentler chi-square difference test [87] was used to compare models. The significance test of the indirect effect of mothers’ individual distress on children’s depression via parenting stress, which would provide crucial evidence of mediation, was based on a bootstrapping procedure (95% confidence intervals with 5000 bootstrap samples). This procedure is recommended for tests of mediation, as it does not require normality of the sampling distribution of the indirect effects [88, 89].

Finally, to test the possible moderation effects of children’s biological sex and age, a multi-group SEM approach was employed, as suggested by several authors [90–92]. In particular, we aimed at testing whether children’s biological sex and age exerted a moderating effect on the entire mediated model. As the moderation effect was to be tested on the entire model, the preferred analytical technique was multi-group analysis (MGA) [91, 92]. MGA tests for structural invariance across groups and compares the effect of every structural path across groups. The invariance of the proposed model was tested separately for: (a) child gender and (b) child age (for a similar approach, see also [93, 94]). In SEM, equivalence across groups can be evaluated by means of constraints that impose identical estimates for model parameters. Specifically, “such tests are conducted by imposing cross group equality constraints on the corresponding parameter estimates for the structural model and then comparing the relative fits of the model so constrained with that of the model without equality constraints. If the fit of the constrained model is not much worse than that of the unrestricted model, there is evidence for structural invariance” [78: p. 420].

Therefore, in the first run, the appropriate structural parameters were freely estimated across groups: this model represented the baseline model. In the second run, the structural parameters were constrained as equal across groups: this model represented the invariant model. The chi-square difference test was used to compare the fit of the two nested models [87]. If the parameters were equal across groups, then the χ2 difference would be not significant and the invariant model should be retained because it would be more parsimonious. In this case, no moderation would occur, because the parameters would not differ across biological sex or age. In contrast, if the chi-square difference between the invariant and baseline models emerged as significant, this would mean that the invariant model fit significantly worse. In this case, parameters would not be equal across the groups and moderation would occur, because the structural parameters differed across biological sex and age. A detailed description of this procedure is also presented in [92] and [93].

## Results

### Correlations and One-Sample t-Test

In the computation of correlations among variables (Table 1), mothers’ individual distress showed a positive association with mothers’ parenting stress and children’s depression. Furthermore, mothers’ parenting stress was positively correlated with children’s depression. Finally, mothers’ individual distress was positively related to mothers’ age and negatively related to children’s age.

**Table 1:** Correlations among variables

	1	2	3	4	5	6
1. Mothers’ Age	1					
2. Children’s Age	0.58**	1				
3. Children’s Biological Sex	− 0.05	− 0.12	1			
4. Mothers’ Mental Distress	− 0.16*	− 0.22**	− 0.02	1		
5. Parenting Stress	0.01	− 0.08	0.03	0.47**	1	
6. Children’s Depression	0.00	0.01	− 0.14	0.39**	0.48**	1

Biological sex was coded as 0 = girls and 1 = boys

*p < 0.05

**p < 0.01

Table 2 presents the means and standard deviations for each study variable compared to a normative mean via one-sample t-tests. All study variables emerged as significantly higher than the normative means.

**Table 2:** Comparison with normative mean

Scales	Sample Mean (SD)	Normative mean	One sample student t	Mean difference	Bootstrap 95% CI	*p* value when compared with normative mean
Mother’s mental distress (HADS)	15.09 (7.57)	13.38	3.24	1.71	[0.67; 2.75]	0.001
Parenting stress (P-CDI)	25.15 (8.90)	19.95	8.39	5.2	[3.98; 6.42]	< 0.001
Children’s depression (PROMIS)	28.15 (10.63)	26	2.90	2.15	[0.69; 3.61]	0.004

df = 205 for all analyses

### Hypothesized Mediational Model

It was hypothesized that the effect of mothers’ individual distress on children’s depression would be mediated by mothers’ parenting stress. The full mediational model displayed a satisfactory fit chi-square (52) = 113.74, *p* < 0.001, RMSEA = 0.08, CFI = 0.96, SRMR = 0.07. However, the partial mediational model in which mothers’ individual distress had a significant direct effect on children’s depression showed a better fit chi-square (51) = 107.67, *p* < 0.001, RMSEA = 0.07, CFI = 0.96, SRMR = 0.06, as the chi-square difference test was significant, chi-square_diff_ (1) = 6.07, *p* = 0.01.

The decomposition of the effects revealed that the indirect effect of mothers’ individual distress on children’s depression via mothers’ parenting stress was significant, mediating approximately 50% of the total effect (Table 3). However, a significant direct effect of mothers’ individual distress on children’s depression remained (Table 3).

**Table 3:** Total and indirect effect

Decomposition of effects	Effect	SE	Bootstrap 95% CI	*p*
Total effect	0.42	0.07	[0.28; 0.54]	< 0.001
Indirect effect: X → M → Y	0.21	0.05	[0.11; 0.32]	< 0.001
Direct effect: X → Y	0.20	0.08	[0.04; 0.37]	0.01

**Fig. 1:**
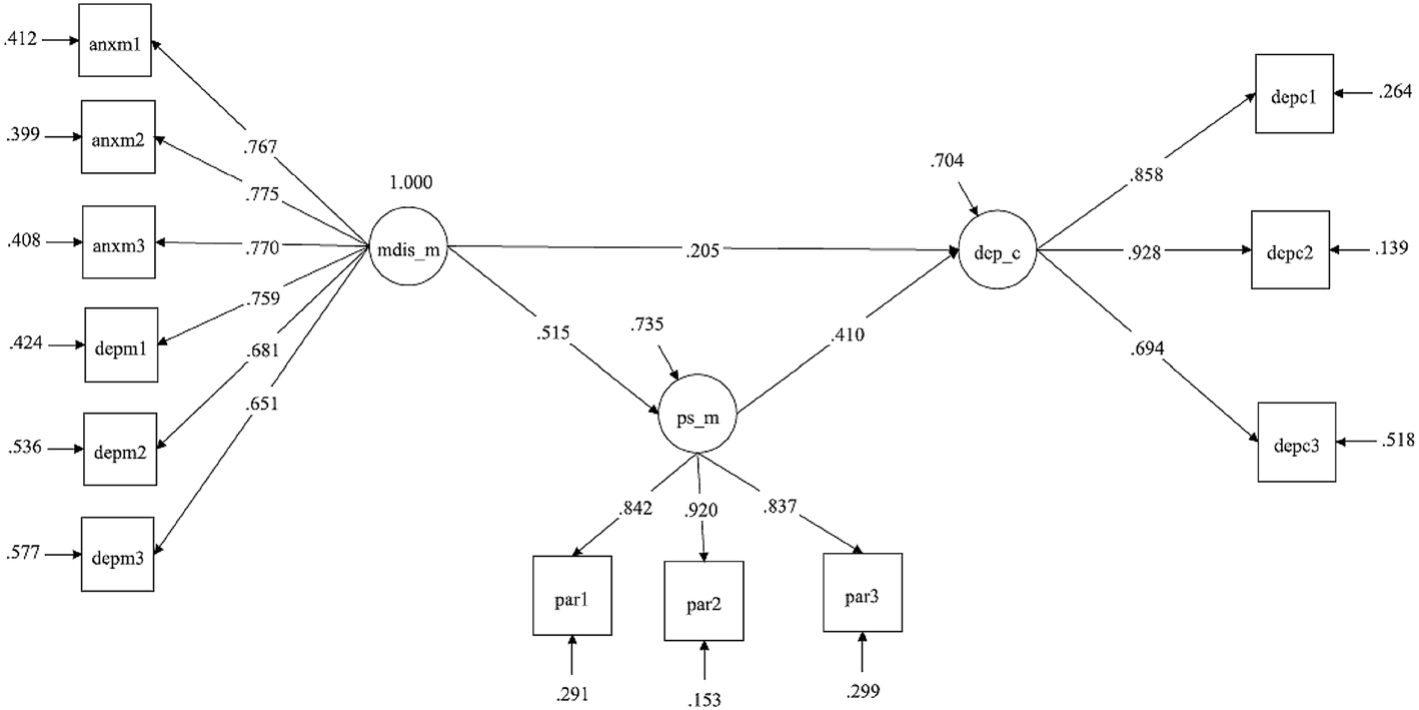
Path analysis with latent variables. standardized coefficients are reported with standard errors between brackets; all coefficients are significant for *p* < 0.01; FIT: Chi-square (52) = 107.67; RMSEA = 0.07; CFI = 0.96; SRMR = 0.05. *mdis_m* mental distress of mothers, *ps_m* parenting stress of mothers, *dep_c* depression of children

As can be noted in Fig. 1, mothers’ individual distress had a significant effect on both mothers’ parenting stress (beta = 0.51, *p* < 0.001) and children’s depression (beta = 0.20, *p* = 0.01), while mothers’ parenting stress was also significantly associated with children’s depression (beta = 0.41, *p* < 0.001). As mentioned, the effect of mothers’ individual distress on children’s depression was mediated by mothers’ parenting stress (indirect effect of = 0.21, *p* < 0.001). Measurements and structural coefficients are fully reported in Fig. 1.

### Multi-Group Analysis

To test for possible moderation effects of children’s biological sex and age on the entire path model, two multi-group analyses were performed to detect model invariance across groups, following the procedure described in the previous section. Table 4 reports the results of the multi-group analyses.

**Table 4:** Multi-group analyses for children’s biological sex and age

	χ^2^	*p*	χ^2^_diff_
Children’s biological sex			
Model 1: baseline, parameter freely estimated	χ^2^(120) = 196.95	< 0.001	
Model 2: invariant, structural parameter constrained equal	χ^2^(124) = 205.59	< 0.001	
			χ^2^_diff_ (4) = 8.64; *p* = 0.07
Children’s age			
Model 1: baseline, parameter freely estimated	χ^2^(120) = 210.34	< 0.001	
Model 2: invariant, structural parameter constrained equal	χ^2^(124) = 213.36	< 0.001	
			χ^2^_diff_ (4) = 3.02; *p* = 0.55

Regarding children’s biological sex, the invariance of the structural coefficients of the partial mediational model was tested and compared with the baseline model, showing that the invariant model could not be rejected, chi-square_diff_ (4) = 8.64, *p* = 0.07 (Table 4). Structural coefficients were invariant across males and females and children’s biological sex did not appear to be a significant moderator. Regarding age, the invariance of the structural coefficients of the hypothesized model was tested across two age groups (7–12 vs. 13–18 years). The results showed that the model was invariant across age, chi-square_diff_ (4) = 3.02, *p* = 0.55 (Table 4). Therefore, children’s age did not appear to moderate the mediational effects tested in our model.

## Discussion

The global impact of the COVID-19 pandemic is continuing to unfold, and it is likely to be felt for years. In response to the pandemic, child environments (e.g., family, school, society) and interactions immediately changed to adjust to new and unexpected scenarios, increasing the risk for mental health problems, in both children and families [17]. Bronfenbrenner’s socioecological model [3] may provide a useful theoretical framework for understanding these particular effects of COVID-19 on mental health. In line with this model, the present study aimed at investigating mothers’ and children’s mental health (i.e., microsystem level) during the first peak of the COVID-19 pandemic in Italy (i.e., macrosystem level), during the government-imposed nationwide lockdown. While this lockdown (i.e., macrosystem level) was necessary to protect the physical health of Italian citizens, it likely had several knock-on effects on the population’s psychological health (i.e., microsystem level).

The present findings shed light on the adjustment of and relationship between mothers and children during this critical period, as most prior studies on this topic have focused on the general population, or only parents’ well-being [8, 95–98]. The first aim was to evaluate mothers’ distress and children’s depression during the lockdown. As for mothers, the collected data showed that individual distress and parenting stress were higher than that of the normative sample; children’s depressive symptoms were also significantly higher than those in the normative sample. Due to the correlational nature of the study, no causal relationships could be inferred; however, the findings suggest an association between the COVID-19 lockdown and a worsening of mothers’ and children’s mental condition. Importantly, children’s mental health was self-reported by children, themselves, and not evaluated by mothers.

During the lockdown, children had to stay at home at all hours of the day. This resulted in disrupted life rhythms, reduced physical activity, and limited or no connection with classmates. It is reasonable to assume that this exceptional situation might have impacted children’s well-being. Previous research on the current outbreak has highlighted that children have not been immune to the dramatic impact of the COVID-19 epidemic; rather, they have experienced physical and social isolation, fear, and uncertainties [54]. In fact, recent studies have shown that a significant proportion of children suffered from mental health disturbances during the lockdown periods in China [99, 100] and Bangladesh [101]. To the best of our knowledge, the present study was the first to directly focus on children in Italy.

The mothers who participated in our research showed higher levels of individual distress and parenting stress than the normative sample. This is understandable, given that the containment measures adopted by the Italian government during the lockdown placed exceptional demands on parents in several areas, including work (forcing parents to cope with job losses or salary reductions, or to transition to smart-working), parenting (pushing parents to manage one or more children at home, independently), family relationships (challenging parents to, e.g., care for their own parents, who may be elderly and sick), intimate relationships with friends and colleagues, and household commitments. Thus, it is not surprising that mothers’ distress increased during this period; indeed, this finding is consistent with previous studies analyzing parental mental health during the lockdown [8, 95–98].

A second aim of the study was to test whether the effect of mothers’ individual distress on children’s depression was mediated by mothers’ parenting stress. Our data confirmed this hypothesis. Parenting stress has classically been defined as parents’ negative experience, resulting from a disparity between perceived parental demands and available parenting resources [11, 12]. As claimed by Crnic and Low [102], parenting stress is a universal experience for parents across all sociodemographic groups and contexts. Nonetheless, high levels of parenting stress may affect the quality of the parent–child relationship, resulting in less optimal parenting [22]. Our findings highlighted that mothers’ individual distress was associated with mothers’ parenting distress, which, in turn, was related to children’s depression. This result is particularly significant in the context of the COVID-19 pandemic, as it highlights the important role played by mothers in buffering the negative effects of the outbreak on their children’s psychological adjustment. As written above, the pandemic and consequent lockdown directly affected children’s well-being by abruptly changing their lives in unforeseen ways. This extreme situation was caused by an invisible enemy (the SARS-CoV-2 virus), which threatened their environment at several levels, including their family, school, and friendship circles. Indeed, research has shown that children’s mental health has been directly affected by the COVID-19 pandemic [54, 99, 100, 103]. Building on this, our results also suggest an indirect association between children’s adjustment and mothers’ individual distress. This finding should be taken into account when developing interventions to support children during a pandemic situation. As stated by Stone et al. [26], parents are the most important environmental factor in the development of psychological problems among children. If, as mentioned, all parents experience parenting stress to some degree [102, 104], this aspect must be a central research interest, especially during the current pandemic. As suggested by Henderson et al. [2], the socioecological model may provide a guiding framework for pediatricians and psychologists to assess, intervene, and support children by identifying the needs of all family members experiencing mental health difficulties due to the COVID-19 outbreak and its consequences. For example, with respect to the microsystem level, professionals may assess children’s mental health issues related to isolation, support parents (especially mothers) through listening, and encourage children and parents to maintain social connections to promote well-being. Furthermore, considering the association between mothers’ adjustment and child development, healthcare professionals might also support children and families remotely during a lockdown situation by teaching stress management skills and parenting efficacy strategies.

We also found that mothers’ individual distress and parenting stress were directly associated with children’s depression. This finding aligns with a recent study of Spinelli and colleagues [8], who found that parents’ stress (both individual and dyadic) related to the COVID-19 emergency increased children’s psychological problems. It could be hypothesized that mothers with higher distress find it more difficult to be available to and to sensitively respond to their children. This problematic interaction may be exacerbated by the extreme situation produced by the COVID-19 pandemic, when distressed mothers may feel even more overwhelmed and may struggle to find appropriate ways to support their children and address their questions and concerns [8]. When children face unresponsive and insensitive mothers, they may be more likely to show greater distress, as evidenced by the high levels of depression in the present sample. From a clinical perspective, this finding suggests that psychological interventions for mothers might also promote children’s well-being. Indeed, parents have been found to play an important role in buffering their children’s stress, helping them to manage their feelings when undergoing difficult experiences [105]. During a pandemic, it is very likely that this parental ability may decrease over time (due to parents’ personal struggles), risking enduring emotional consequences for children. However, more research is needed on this topic, as the cross-sectional nature of the collected data does not allow conclusions to be drawn.

A further aim was to evaluate the moderation effects of children’s biological sex and age in the mediation model. The results of the multi-group analyses showed that neither biological sex nor age were significant moderators, highlighting that the proposed model was robust and invariant across children’s sex and age. This result was consistent with the study of Morelli et al. [96], who found that children’s psychological distress was not affected by age or gender. Furthermore, our multi-group analyses showed that the relationships among variables did not change according to children’s biological sex or age; thus, the entire model was replicated across boys and girls and across children and adolescents. Given this, it is plausible to conclude that the mechanisms involved in the psychological distress of children and adolescents were similar for boys and girls and for children and adolescents, due to the general health emergency situation of COVID-19, which was filtered through parents. Thus, the direction and strength of the relationships among variables did not depend on different levels of child dependence on caregivers related to children’s biological sex or age. This result is in line with the theoretical framework of Bronfenbrenner’s socioecological model [3], stating that children’s psychological well-being and functioning strictly depend on the context in which they live.

Overall, our findings highlighted the importance of considering mothers’ distress—both individual and dyadic—as it might affect their interactions with children. Indeed, mothers with higher levels of stress may have found it more difficult to understand and sensitively respond to their children’s needs [106]. In response, the children who interacted with such mothers may have felt less understood, which might have increased their negative feelings beyond the level that had already been activated by the lockdown measures.

Some limitations of this study must be taken into account. First, the use of self-report questionnaires did not allow us to draw clinical diagnoses. Future research should include participants with high levels of depression or distress based on standardized interviews, in order to determine whether they meet the criteria for a clinical disorder. Moreover, the cross-sectional nature of the research did not allow conclusions to be drawn about causal relationships. Future longitudinal studies could address this issue. Furthermore, we cannot exclude the possibility that child behavior affected parenting stress, as this was not considered in the analyses; however, this issue was previously examined by Stone et al. [26], who did not find an effect of children’s internalizing problems on parenting stress. Additionally, we obtained an acceptable response rate of 76.74% amongst all of the mothers reached. We then compared the mothers who did and did not give consent for their children to participate, finding no significant differences between groups with respect to any of the investigated variables. Among the mothers who consented for their child to participate, we achieved a response rate of 53.37%, which may be considered only moderate. While this may have affected the results of the present study, we tested for differences between the mothers whose children filled in the questionnaires and those whose children did not fill in the questionnaires and, again, no significant differences were found. Finally, we collected a convenience sample that was not necessarily representative of the Italian population.

Notwithstanding these limitations, the present study has important implications for at least two reasons: methodologically, children’s adjustment was not evaluated by mothers (as is typical), but directly by the children themselves, thereby reducing any biases associated with one-informant procedures; and theoretically, the study shed light on the mediational role played by parenting stress in the relationship between mothers’ mental health and children’s depression. Our data suggest some relevant practical considerations for promoting well-being and preventing the onset of depressive symptoms in children. As is well known, children have fewer personal resources than adults when dealing with the significant life changes produced by a pandemic [107]; thereby, in this situation, parents constitute their primary reference, especially when all other adult figures (i.e., teachers, grandparents, coaches) are absent due to social distancing regulations. In the current and future pandemics, public health services should support parents—and particularly mothers—in reducing both individual distress and parenting stress, not only through online counseling services, but also through guidelines on how to communicate with children about the pandemic. Furthermore, the European Pediatric Association–Union of National European Pediatric Societies and Associations (EPAUNEPSA) [54] recently underlined the protective role of parents in reducing children’s fear and stress [96]. While it is understandable that, during the initial phase of the COVID-19 outbreak, the government focus was on citizens’ physical health, the time is now ripe to increase attention on psychological health, also considering that pandemics may become more frequent in the future.

## Summary

The present study, carried out during the first peak of the COVID-19 outbreak in Italy, aimed at investigating the mental health of mothers and children during the nationwide lockdown. A total of 206 Italian mothers and their children completed an online survey. Mothers were administered self-report questionnaires to measure individual distress and parenting stress; children completed a standardized measure of depression. The results showed that mothers’ individual distress and parenting stress were higher than those displayed by the normative sample; also, children’s depressive symptoms were significantly higher than those of the normative sample. Another important finding was that mothers’ parenting stress mediated the association between mothers’ individual distress and children’s depression. Specifically, our data highlighted that mothers’ individual distress was associated with parenting distress that, in turn, impacted children’s depressive levels. This result is particularly significant in the context of the COVID-19 pandemic, as it highlights the important role played by mothers in buffering the negative effects of the outbreak on children’s psychological adjustment. The study has important implications for at least two reasons: methodologically, children’s adjustment was not evaluated by mothers (as is typical), but directly by the children themselves, thereby reducing any biases associated with one-informant procedures; and theoretically, the study shed light on the mediational role played by parenting stress in the relationship between mothers’ mental health and children’s depression.

These findings must be taken into account when designing interventions to support mothers and children during emergency situations.
